# Colistin-Resistant *Escherichia coli* Isolated from Houseflies and Feces of Cattle and Pigs at a Slaughterhouse in Lima, Peru

**DOI:** 10.3390/antibiotics14080818

**Published:** 2025-08-10

**Authors:** Andrea Carhuallanqui, Lorena Villafana, Rosa Gonzalez-Veliz, José F. Cobo-Díaz, Avelino Álvarez-Ordoñez, Daphne Doris Ramos-Delgado

**Affiliations:** 1 Public Health and Environmental Health Laboratory, Universidad Nacional Mayor de San Marcos, Lima 15081, Peru; acarhuallanquipe@unmsm.edu.pe (A.C.); lorena.villafana@unmsm.edu.pe (L.V.); 2 Avian Pathology Laboratory, Universidad Nacional Mayor de San Marcos, Lima 15081, Peru; rgonzalezv@unmsm.edu.pe; 3 Department of Food Hygiene and Technology and Institute of Food Science and Technology, Universidad de León, 24004 León, Spain; jcobd@unileon.es (J.F.C.-D.); aalvo@unileon.es (A.Á.-O.)

**Keywords:** colistin, *Musca domestica*, pig, cattle, *Escherichia coli*, antimicrobial resistance genes

## Abstract

**Background:** Pigs and cattle have been implicated as reservoirs of antimicrobial resistance genes (ARGs) that can spread to humans, and houseflies are considered potential carriers of bacteria with ARGs that could contribute to their spread to the environment, including food, animals, and humans. **Methods:** In this study, 107, 145, and 127 *Escherichia coli* strains were isolated from houseflies, pigs, and cattle, respectively, from a slaughterhouse in Lima, Peru. Antimicrobial susceptibility testing was performed using the Kirby–Bauer method, where thirteen antibiotics were used. Strains were also plated on CHROMagar COL-APSE agar, and colistin’s minimum inhibitory concentration (MIC) was determined. Colistin-resistant *E. coli* strains were subjected to whole genome sequencing. **Results:** 7.8% (8/107), 1.38% (2/145), and 0.79% (1/127) of *E. coli* strains isolated from houseflies, pigs, and cattle, respectively, were resistant to colistin (MIC ≥ 4 µg/mL). ARGs associated with resistance to more than 6 different antibiotic classes were identified, including tetracyclines, beta-lactams, fluoroquinolones, nitroimidazoles, trimethoprim and amphenicols. **Conclusions:** This study suggests that flies could contribute to the dissemination of ARG carrying bacteria and shows the potential risk of animals and meat production systems as reservoirs of ARG carrying bacteria.

## 1. Introduction

The spread of antibiotic-resistant bacteria has become a multi-sectoral and multi-factorial global problem, causing at least 700,000 deaths per year worldwide, and is estimated to reach ten million deaths by 2050 [1]. The misuse and overuse of antibiotics in animal agriculture, particularly as growth promoters, prophylaxis, and metaphylaxis, has significantly contributed to the emergence and spread of antimicrobial resistance, with implications for human and environmental health [2].

Colistin, or polyxin E, is considered a last-resort antibiotic for the treatment of multidrug-resistant Gram-negative bacterial infections in humans [3]. However, it was widely used in animal agriculture for the prevention and treatment of infections caused by *Enterobacteriaceae* and as a growth promoter in animal feed for human consumption, making its resistance a concern [4,5].

Colistin resistance can be caused by efflux pumps, loss of lipid A, and the decrease in the net negative charge of the bacterial outer membrane due to the addition of cationic groups (L-Ara4N and pEtN) to lipopolysaccharide [6]. Genetic determinants responsible for colistin resistance include chromosomal mutations in the genes *mgrB*, *prmE*, *pmrAB*, *phoQ, arnT,* or *eptA* [7,8,9]. The first plasmid-mediated colistin resistance gene (*mcr-1*) present in *Escherichia coli* was reported in 2015 [10] in an isolate from China, and nine *mcr* homologs have been reported since then [11]. Horizontal transfer of the *mcr-1* gene has emerged as a significant contributor to the dissemination of colistin resistance among various Gram-negative bacteria [4,12]. The global spread of the *mcr-1* gene suggests that the use of colistin in veterinary medicine has likely accelerated the spread of this gene between humans and animals [13]. Ten different variants of the mcr gene have been reported worldwide (*mcr-1* to *mcr-10*) in clinical and environmental settings, representing a significant public health problem [12,14].

The housefly (*Musca domestica*) is a synanthropic insect with a worldwide distribution found in homes, restaurants, slaughterhouses, and animal production environments [15,16]. Furthermore, it comes into direct contact with contaminated areas, such as landfills and wastewater; therefore, it can be a vehicle for the transport of bacteria to animals and humans [15]. When feeding on contaminated fluids, the fly can pick up bacteria and these bacteria can multiply in their digestive tract [17]. As a result, houseflies can transmit pathogenic bacteria through regurgitation, mechanical translocation, and defecation [18,19]. Studies have shown that houseflies can be potential vectors of antimicrobial-resistant pathogenic *Enterobacteriaceae* in agricultural and slaughterhouse settings, posing a risk for transmission of resistant bacteria to the environment, animals, and humans [20,21,22,23].

Few studies have been conducted in Peru on antibiotic resistance in enterobacteria isolated from houseflies [19]. This study aims to pheno- and geno-typically characterize colistin-resistant *E. coli* isolated from houseflies and feces of cattle and pigs at slaughterhouses in Lima. Additionally, it seeks to expand and strengthen knowledge of antimicrobial resistance in Peru.

## 2. Results

All *E. coli* strains were confirmed by biochemical testing, and a total of 379 *E. coli* strains were obtained. A total of 107, 145, and 127 *E. coli* strains were isolated from houseflies, pigs, and cattle, respectively. Of the *E. coli* isolates from houseflies, pigs, and cattle, 7.48% (8/107), 1.38% (2/145), and 0.79% (1/127), respectively, were resistant to colistin.

### 2.1. Antimicrobial Susceptibility Testing

One-hundred-and-seven strains of *E. coli* were isolated from house flies, and resistance to lincomycin (100%), tetracycline (90.65%), ampicillin (74.77%), amoxicillin (72.90%), and chloramphenicol (69.16%) was reported. (Table 1). One-hundred-and-forty-five strains of *E. coli* were isolated from pig feces, and resistance to lincomycin (100%), tetracycline (97.24%), chloramphenicol (92.41%), ampicillin (89.66%), and sulfamethoxazole-trimethoprim (77.93%) was reported (Table 1). One-hundred-and-twenty-seven strains of *E. coli* were isolated from cattle feces, and resistance to lincomycin (100%), tetracycline (81.89%), chloramphenicol (22.05%), amoxicillin (20.47%), and ampicillin (18.90%) was reported (Table 1).

Pig feces showed a significantly higher prevalence of multidrug-resistant *E. coli* strains (93.73%) than *E. coli* strains isolated from house flies (71.96%) (*p* < 0.001). Likewise, flies and pig feces showed a significantly higher prevalence than *E. coli* strains isolated from cattle (19.68%) (*p* < 0.001). The multidrug-resistant *E. coli* strains were resistant to ampicillin, chloramphenicol, sulfamethoxazole-trimethoprim, ciprofloxacin, tetracycline, and gentamicin.

### 2.2. Colistin Resistance

The 7.48% (8/107), 1.38% (2/145), and 0.79% (1/127) of the *E. coli* isolates from houseflies, pigs, and cattle, respectively, were resistant to colistin (MIC ≥ 4 µg/mL); and *mcr-1* gene was not detected, by PCR, in these isolates. The eleven colistin-resistant *E. coli* strains were multidrug-resistant (Table 2), and their complete genomes were sequenced, employing the following codes for the genomes labeling: pig (203B and 150B), houseflies (107M, 84M, 110M, 61M, 81M, 95M, 21M, and 65M) and cattle (131V).

### 2.3. Serotype and Multilocus Sequence Typing (MLST)

**Figure 1:**
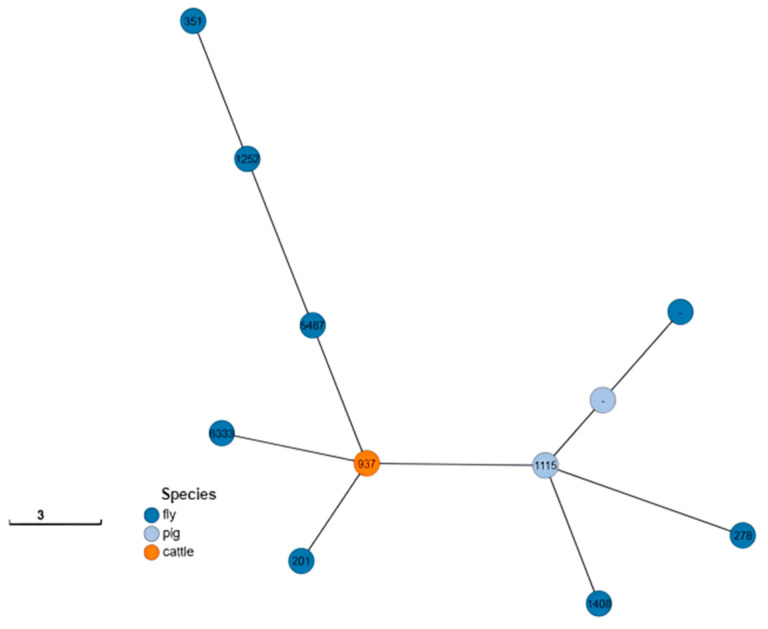
GrapeTree based on MLST scheme of 11 *E. coli* genes according to species of origin and sequence type [1,2,8].

Nine *E. coli* strains were typed considering serogroup O, whereas two *E. coli* strains isolated from flies were unassignable (non-typeable serogroup O). All 11 strains were assigned to a known serogroup H. Overall, ten different H types and six O types were identified (Table 3). Achtman’s seven-locus MLST scheme was used, and nine different sequence types (STs) were identified, but two *E. coli* strains (from flies and pigs) could not be assigned to known STs (Table 3; Figure 1).

### 2.4. Virulence Genes

One *E. coli* strain isolated from flies had the yuA gene, and one *E. coli* strain isolated from pigs had the ironN gene, both genes associated with iron acquisition systems. The *E. coli* strain isolated from bovine feces presented the f17d-G gene (fimbrial-type adhesin). All strains presented the fimH gene (fimbria type 1 adhesin) and the ompA gene (serum resistance) (Table 4). No major virulence determinants associated with *E. coli* pathotypes, such as hlyA, stx, elt, eae, aggR, ipaH, and hlyA genes, were detected.

### 2.5. Antimicrobial Resistance Genes and Chromosomal Gene Mutations Associated with Antimicrobial Resistance

**Figure 2:**
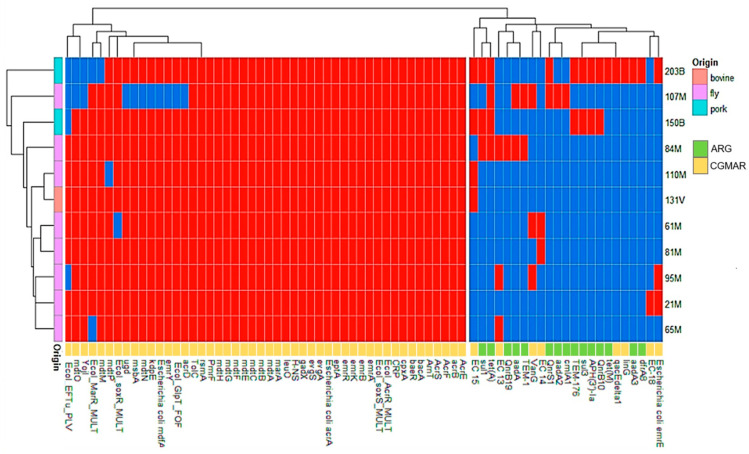
Heatmap (presence/absence) of acquired antimicrobial resistance genes (ARGs) and chromosomal gene mutations associated with antimicrobial resistance (CGMAR) of *E. coli* isolated from flies, pigs, and cattle. Red squares indicate presence and blue squares absence of ARG/CGMAR.

ARGs associated with resistance to seven different antibiotic classes were identified: aminoglycosides (*aadA3*, *aadA*, *aadA2* and *aph(3’)-la*), tetracyclines (*tet(A)*, *tet(M)*), beta-lactams (*bla_TEM-1_*, *bla_TEM-176_*), fluoroquinolones (*qnrs1*, *qnrsB10* and *qnrB19*), trimethoprim (*dfrA8*), sulfonamides (*sul1* and *sul3*), and amphenicols (*cmlA1*). In addition, more than fifty point mutations in intrinsic chromosomal genes associated with antimicrobial resistance were identified (Figure 2).

The 11 *E. coli* strains did not present mobile colistin resistance genes; however, all genomes contained chromosomal point mutations previously linked to colisin resistance (*arnT*, *eptA*, and *pmrF*).

**Figure 3:**
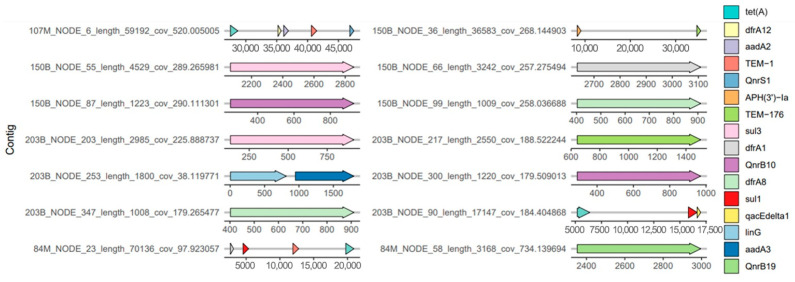
Plasmidic contigs with resistance genes from colistin-resistant *E. coli* strains isolated from pigs and houseflies (107M, 84M: housefly; 150B, 203B: pig).

Fourteen plasmidic contigs with ARGs were identified in four *E. coli* strains: 107M (1 plasmidic contig), 84M (2), 150B (5), and 203B (6). They harbored *tet(A)*, *dfrA12*, *dfrA1*, *dfrA8*, *aadA2*, *aadA3*, *bla_TEM-1_*, *bla_TEM-176_*, *qnrS1*, *qnrB10*, *qnrB19*, *APH(3′)-Ia*, *sul3*, *sul1*, *qacEdelta1*, and *linG* (Figure 3).

### 2.6. Phylogenetic Analysis

**Figure 4:**
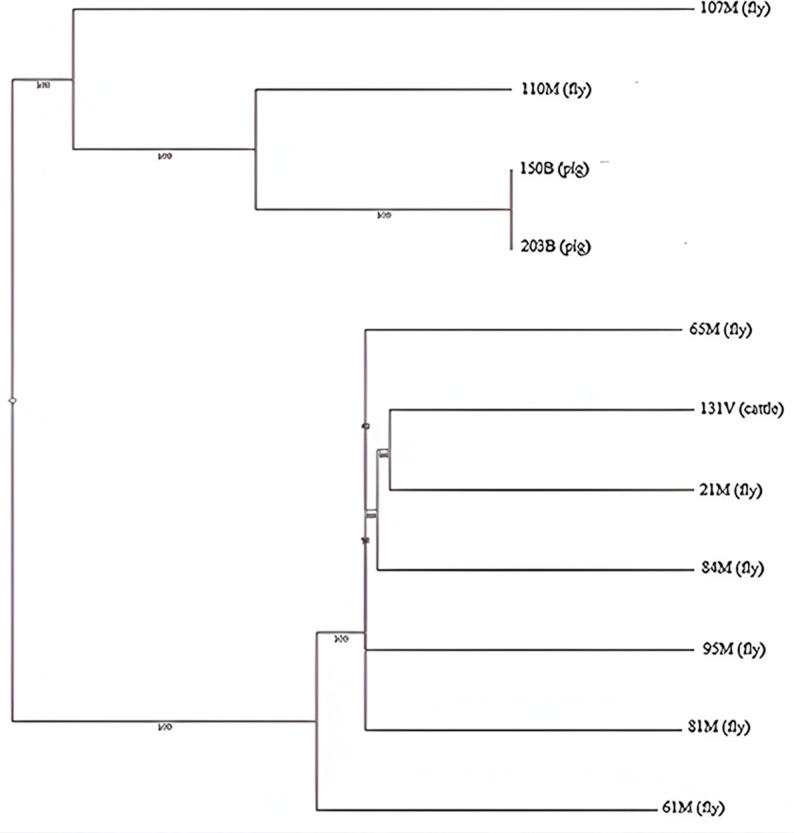
cgMLST phylogenetic analysis of genomic sequences obtained from *E. coli* strains isolated from flies, cattle and pigs.

The genomes were grouped into two clusters: 107M (fly), 110M (fly), 150B (pig), and 203B (pig), representing the first cluster, and 65M, 61M, 21M, 84M, 95M, and 81M (flies), and 131V (cattle), representing the second cluster. cgMLST analysis revealed a shared ancestry between the single *E. coli* genome isolated from cattle (131V) and an *E. coli* genome isolated from a fly (21M). Furthermore, it revealed a common ancestry between two *E. coli* genomes isolated from pigs (150B and 203B) and one *E. coli* genome isolated from a fly (110M) (Figure 4).

## 3. Discussion

In the present study, a total of 379 strains of *E. coli* were isolated from domestic flies (107), pigs (145), and cattle (127). 93.73%, 71.96%, and 19.68% of the *E. coli* strains isolated from pigs, flies, and cattle, respectively, were multidrug-resistant (to ampicillin, chloramphenicol, sulfamethoxazole-trimethoprim, ciprofloxacin, tetracycline, and gentamicin) (Table 1). This study demonstrates that asymptomatic cattle and pigs slaughtered at a slaughterhouse in Lima carry *E. coli* resistant to various antibiotics. *E. coli* strains isolated from cattle feces showed significantly lower multidrug resistance than *E. coli* strains isolated from pigs and flies. The difference between cattle and pigs is due to the type of livestock system and stocking density in both production systems. Cattle are generally raised in an extensive livestock system, while pig farming is an intensive livestock system. Antimicrobials are routinely used to prevent disease in intensive systems, contributing significantly to the emergence of antimicrobial resistance in pigs [24,25]. Furthermore, flies present in the slaughterhouse are in constant contact with animal feces, which can spread resistant bacteria.

International guidelines on the use of antibiotics as growth promoters vary across countries. European countries and the US have banned antibiotics as growth promoters in animal feed [26,27,28]. China also adopted this legislation in 2020, banning 11 antibiotics [29]. Growth promoters based on all antibiotic classes for all animal species have been banned in Chile and Colombia; Argentina only allows mixing certain antibiotics, none of which are critically important for human medicine; Brazil has been phasing out many antibiotic classes; Uruguay prohibits using antibiotics as growth promoters in sheep and cattle, but no legislation was found regarding their use in pigs and chickens [30]. In contrast, Peru does not have legislation prohibiting antibiotics in growth promoters; only antibiotics such as chloramphenicol, nitrofurans, olaquindox, nitroimidazoles, and colistin have been banned.

The Kirby–Bauer method for antimicrobial resistance profiling revealed a higher percentage of resistance to most antibiotics in *E. coli* strains isolated from pigs compared to those from cattle. Globally, antimicrobial consumption is estimated to be higher in pigs compared to cattle [31,32]. Subtherapeutic antibiotics have been used for many years in food production animals to control the spread of infectious diseases among crowded animals and improve production performance, especially in pig and poultry farming. In pig production, antibiotics can be applied to entire groups by mixing them in feed and water [33].

The resistance profile, analysed using the Kirby–Bauer method, of the *E. coli* strains isolated from cattle and pig feces showed a tetracycline resistance of 81.89% and 97.24%, respectively. Studies in Latin America also reported high percentages of tetracycline-resistant *E. coli*. Melgarejo-Touche et al. [34] evaluated antimicrobial resistance in stool samples from beef cattle slaughtered in *Asunción* (Paraguay) slaughterhouses and reported that 100% of fluoroquinolone-resistant *E. coli* were resistant to tetracycline. Cabrera González et al. [35] evaluated the resistance of *E. coli* isolated from the diarrheal feces of newborn calves in the Cajamarca region (Peru) and they found resistance to tetracycline (96.15%), sulfamethoxazole-trimethoprim (51.92%), neomycin (26.92%), and enrofloxacin (9.61%).

The use of tetracyclines as feed additives and drugs for animals intended for human consumption is not forbidden in Peru and it may be the main cause of the high percentage of tetracycline-resistant *E. coli* strains. The use of tetracyclines in veterinary medicine is common due to several advantages, including low cost, oral administration, and broad spectrum of activity [36]. Genes associated with tetracycline resistance were found in colistin-resistant *E. coli* strains isolated from pigs *(tet(A)*, *tet(M)*); point mutations in the genes *emrK*, *emrY*, *evgA*, and *evgS* were also detected in *E. coli* strains isolated from pigs and cattle. *tet* genes generally cause tetracycline resistance and, in the case of Gram-negative bacteria such as *E. coli*, encode efflux pump systems [36]. *tet* genes were found in the two *E. coli* strains isolated from pigs and two *E. coli* strains isolated from flies (84M and 107M). *tet(A)* was found on three plasmids in *E. coli* isolated from houseflies (2) and pigs (1). Therefore, the high frequency may also be due to the horizontal transfer of these genes, as has been reported recently [37].

All *E. coli* strains isolated from pigs, cattle, and flies were resistant to lincomycin; this may be due to cross-resistance with macrolides and group B streptogramin (MLSB) caused by ribosomal modification by methylases encoded by *erm* genes [38,39]. The 11 colistin-resistant *E. coli* strains exhibited point mutations in the *erm(A)* and *erm(B)* genes, conferring resistance to lincomycin. This resistance could be due to the use of macrolides as growth promoters in cattle and pigs [40]. On the other hand, most *E. coli* strains isolated from flies were resistant to ampicillin (74.77%) and tetracycline (90.65%). The percentage of resistance detected could be a consequence of the high consumption of these antimicrobials in pig and beef cattle production [35,40,41]. Other studies also reported that enterobacteria isolated from house flies were frequently resistant to tetracycline and ampicillin [42,43,44].

The Peruvian Ministry of Agriculture and Irrigation (MINAGRI) banned the import, marketing, and manufacture of active ingredients of chloramphenicol, nitrofurans (furazolidone and nitrofurantoin), olaquindox and nitroimidazoles in Peru in 2013 and in 2019 colistin was banned for use in animals [45]. However, despite the ban, colistin-resistant bacteria have been reported in animals for human consumption [41]. Chloramphenicol resistance possibly originated through mutation of the *acrA*, *acrB* and *tolC* genes because the AcrAB efflux system belongs to the nodulation-division transporter (NDT) family and uses the TolC system. Substrates for this efflux system (AcrABTolC) have included chloramphenicol, tetracyclines, fluoroquinolones, and trimethoprim [46,47]. In addition, this study reported resistance to nitrofurantoin, using the Kirby–Bauer method, in *E. coli* strains isolated from pigs (1.38%) and flies (3.74%). However, in the 11 colistin-resistant *E. coli* strains, no genes associated with nitrofurantoin resistance were found.

Phylogenetically, eleven colistin-resistant *E. coli* strains belonged to group A (3/11) and B1 (8/11), being classified as non-pathogenic commensal strains, representing a reduced risk to the environment and public health [48]. This is consistent with other studies that reported that *E. coli* strains isolated from cattle and pigs most frequently belong to group B1 and/or A [49,50]. The *E. coli* strains isolated from pigs (150B and 203B) were identified to present the same phylogroup as a strain isolated from *M. domestica* (110M). Likewise, *E. coli* strains with the same phylogroup for flies (107M, 21M, 61M, 65M, 81M, 84M, and 95M) as for cattle (131V) were identified.

The eleven isolates sequenced in this study belong to different sequence types, suggesting a diversity in colistin-resistant *E. coli* strains. The use of media not supplemented with colistin eliminated the selective pressure and limited the recovery of colistin-resistant *E. coli*. This work revealed a diverse resistome with ARGs that confer resistance to more than 10 antibiotic classes, including chloramphenicol, tetracycline, aminoglycosides, and ciprofloxacin. These results demonstrate the circulation of colistin-resistant *E. coli* carrying different antibiotic-resistance genes in houseflies and cattle in Lima. This is consistent with projections, which indicate that Latin America is increasing the consumption of antimicrobials by cattle [51].

Plasmids can disseminate antimicrobial resistance genes in Gram-negative bacteria. They generally harbor determinants that confer resistance to different classes of antibiotics simultaneously; in this study, plasmids with resistance genes to tetracyclines, fluoroquinolones, ESBLs, aminoglycosides, lincomycins, trimethoprim, and sulfonamides were found. The following incompatibility plasmids were found: IncFIB (AP001918) (107M, 84M) and IncX1 (150B), and a colicinogenic plasmid in strain 84M (Col(pHAD28)). In addition, the IncX1 plasmid presented the APH genes *aph(3′)-Ia* and *blaTEM-176*; plasmid families that include IncX play an important role in the propagation of ESBL genes [52]. Plasmids IncFIB (AP001918) and Col(pHAD28) were also reported in *E. coli* strains in drinking water and feces of dogs and cattle in rural areas of Cajamarca, Peru [53].

The two genomes of the *E. coli* strains isolated from pigs presented five (150B) and six plasmidic contigs (203B) with antimicrobial resistance genes. The *qacEdelt1* gene was identified in a plasmid of one *E. coli* strain isolated from pigs (203B), together with ESBL genes and genes of resistance to sulfonamides, lincomycin, fluoroquinolones, and tetracyclines. This indicates possible co-resistance to disinfectants such as quaternary ammonium compounds [54,55]. In addition, there are studies where the *qacEdelt1* gene has been considered a genetic marker of class 1 integrons associated with multidrug-resistant phenotypes [56].

There are several antibiotics used in animals for human consumption and classified as critically important antimicrobials for the treatment of people; among them is colistin, which, according to the World Health Organization’s list of critically important antimicrobials for human medicine, is considered among those with the “highest priority” [57]. Only eleven strains of *E. coli* were phenotypically resistant to colistin, and none presented *mcr* genes. The eleven *E. coli* strains presented point mutations in the *arnT* and *eptA* genes that can individually or collectively affect the efficiency of the phosphoethanolamine transferase enzyme, altering the structure of the bacterial membrane and reducing the ability of colistin to bind and exert its antimicrobial effect [9].

The most common mechanism of colistin resistance is due to the mutation of chromosomal genes associated with the modification of lipid A of LPS, the main target of colistin, as an adaptive response [58]. Such modifications can be obtained by the addition of 4-amino-4-deoxy-L-arabinose (L-Ara4N) and phosphoethanolamine (PEtN) to lipid A [59] (Baron et al., 2016). Genes encoding enzymes involved in lipid A synthesis are *pmrHFIJFKLM* (*arnBCADTEF*-*pmrE*). These genes are positively regulated by the two-component systems PhoPQ and PmrAB [9]. Resistance to colistin has been previously linked to mutations in *pmrAB* and *phoPQ* (two-component regulatory systems) [7]. The *arnBCADTEF* operon, which is activated by PmrAB, triggers the synthesis and addition of L-Ara4N to lipid A, reducing the binding affinity to colistin [60]. In addition, *eptA*, which PmrAB also activates, is involved in the synthesis and addition of PEtN; consequently, L-Ara4N and PEtN are added to LPS, decreasing the negative charge of the outer membrane and reducing the binding affinity to colistin [61].

All 11 colistin-resistant strains presented point mutations in the *mdtM* gene. *MdtM* is a monocomponent multidrug efflux protein that belongs to the major facilitator superfamily and interacts with membrane-spanning transporters, such as the *AcrABeTolC* complex, to form a coordinated supersystem of membrane transporters that confers antimicrobial resistance [62]. The mutation of the *mdtM* gene in all *E. coli* strain genomes is likely because its multidrug efflux activity is a co-opted adaptation of its physiological functions; therefore, it persists in the bacterial genome even in the absence of pharmacological selective pressure [63].

Multidrug resistance is most frequently found in pigs, followed by flies, and less frequently in cattle. These *E. coli* strains can be transmitted to humans through the food chain, posing a public health risk. Animal waste can also spread antimicrobial-resistant bacteria into the environment [64]. Furthermore, phylogenetic analysis showed a possible circulation of colistin-resistant *E. coli* between animals in the slaughterhouse and houseflies. This study found that colistin-resistant *E. coli* strains presented plasmids with resistance genes to different antimicrobials; this indicates a significant risk for public health, being able to transmit genes to other Gram-negative bacteria horizontally [65]. The housefly could transmit these bacteria through regurgitation of vomit and defecation. In addition, “bioenhanced transmission” has been reported, where bacteria can multiply at regurgitation sites, mouthparts, and the intestine [18]. From pigs and cattle and through houseflies, these *E. coli* strains could enter the food chain to contaminate food and other environments. The frequency of antimicrobial resistance in pigs reported in the present study highlights the urgent need to restrict and ensure the prudent use of antimicrobials in Peru.

This study showed that houseflies collected inside the slaughterhouse presented *E. coli* strains resistant to different antimicrobials, possibly because the flies easily acquired the bacteria when they fed on the manure and waste found in the slaughterhouse [66]. However, they could also have acquired these bacteria from other environments, as they can fly 5–7 km [67,68,69]. Studies have shown the presence of antimicrobial-resistant enterobacteria isolated from houseflies in different environments [18,19,42,70].

## 4. Materials and Methods

### 4.1. Flies Sampling and Escherichia coli Isolation

One hundred fifty houseflies were collected using an entomological net from pig and cattle pens at a slaughterhouse in Lima, Peru, from February 2022 to October 2023. Five flies were captured every fifteen days. After collection, the flies were placed in sterile jars and immediately transported at 4 °C to the Public Health and Environmental Health Laboratory of the Faculty of Veterinary Medicine of the Universidad Nacional Mayor de San Marcos (FMV- UNMSM). Each fly was morphologically identified using a stereoscope and following the keys of Greenberg [71]. The main steps of the Pava–Ripoll et al. [72] protocol were followed for the dissection of flies; the flies were sacrificed by freezing them to a temperature of −20 °C and then disinfected with 70% ethanol and washed individually in sterile saline solution.

The digestive tracts of the flies were removed with fine-tipped dissecting forceps and placed individually in 5 mL vials containing peptone water. The digestive tract suspensions were homogenized for three minutes and incubated individually at 37 °C for 18 h. After incubation, a 100 μL aliquot was plated on Petri dishes containing eosin methylene blue agar (EMB) (Merck, Darmstadt, Germany). EMB agar plates were then incubated at 37 °C for 24 h. Two suspected *E. coli* colonies from each fly were streaked on Tryptone Soy Agar (TSA, Merck, Germany) for confirmation with conventional biochemical tests, such as plating on Triple Sugar Iron Agar (TSI, Merck, Germany), Lysine Iron Agar (LIA, Merck, Germany), Sulfide Indole Mobility (SIM, Merck, Germany), Urea Broth (Merck, Germany) and Simmons Citrate Agar (Merck, Germany), following the procedures described elsewhere [73,74,75]. All confirmed *E. coli* strains were subjected to antimicrobial susceptibility testing against 13 commonly used human and veterinary medicine antibiotics.

### 4.2. Collection of Feces from Pigs and Cattle and Isolation of E. coli

In addition to house flies, aseptically collected fecal samples were obtained from cattle (n = 150) and pigs (n = 150) in the same slaughterhouse and time as the flies. Sampling was carried out in the abdominal viscera washing area of the slaughterhouse, making an incision in the intestine approximately 65 cm proximal to the anal sphincter [76]. Approximately 150 g of feces were collected from the distal area of the large intestine using a sterile polyethylene bag. Five samples of each species were taken every fifteen days. The samples were transported at 4 °C immediately to the laboratory for processing.

*E. coli* enrichments were prepared by diluting 25 g of feces in 225 mL of buffered peptone water (Merck, Germany). Subsequently, each enrichment was homogenized for 1 min and incubated at 42 °C for 18 h [76]. For the isolation of *E. coli*, 100 µL of peptone water was plated on EMB agar. The EMB plates were incubated at 37 °C for 24 h. For confirmation, two suspected *E. coli* colonies per sample were plated on TSA agar for confirmation with conventional biochemical tests such as TSI agar, LIA agar, SIM agar, urea broth, and Simmons Citrate agar, and the antimicrobial susceptibility of all confirmed *E. coli* isolates was tested, as described above for the housefly *E. coli* isolates.

### 4.3. Antimicrobial Susceptibility Testing

A total of 107, 145, and 127 *E. coli* strains were isolated from houseflies, pigs, and cattle, respectively, and were tested for antimicrobial susceptibility. The Kirby–Bauer disk diffusion method was used on cation-adjusted Mueller Hinton agar (Merck, Germany) [77]. The antibiotic disks used were as follows: amoxicillin (20 μg), ampicillin (10 μg), cephalexin (30 μg), ciprofloxacin (5 μg), chloramphenicol (30 μg), enrofloxacin (5 μg), gentamicin (10 μg), lincomycin (2 μg), nalidixic acid (30 μg), neomycin (30 μg), nitrofurantoin (300 μg), sulfamethoxazole-trimethoprim (1.25/23.75 μg), and tetracycline (30 μg). Results were interpreted according to CLSI guidelines [77].

Moreover, *E. coli* strains were grown on CHROMagar COL-APSE agar (CHROMagar, La Plaine St-Denis, France) at 37 °C to test for colistin resistance. In addition, the minimum inhibitory concentration (MIC) of colisitin was determined by broth microdilution using ComASP™ Colistin (Liofilchem, Roseto degli Abruzzi, Italy) and following the manufacturer’s recommendations. *Escherichia coli* ATCC 25922 and a molecularly characterized *E. coli* strain CP141, carrying the *mcr-1* gene, from the Public Health and Environmental Health Laboratory of FMV-UNMSM were used for quality control of the tests.

### 4.4. Detection of the mcr-1 Gene

The possible presence of *E. coli mcr-1*-positive strains was assessed by conventional PCR in all *E. coli* strains with a colistin MIC > 2 μg/mL. For that, *E. coli* isolates were grown overnight on TSA agar (Merck, Germany), and genomic DNA was extracted using the GeneJET Genomic DNA Purification kit (Thermo Scientific, Waltham, MA, USA), following the manufacturer’s protocol. A NanoDrop One spectrophotometer (ThermoFisher Scientific, USA) was used to determine DNA purity and concentration. Finally, the DNA was stored at −20 °C until further analysis. The PCR analysis was performed using as primers CLR5-F (5′-CGGTCAGTCCGTTTGTTC-3′) and CLR5-R (5′-CTTGGTCGGTCTGTAGGG-3′), as described by Liu et al. [10].

A final reaction volume of 25 µL was used (23 µL of Master Mix and 2 µL of DNA). The Master Mix consisted of 12.5 µL of DreamTaq Green PCR Master Mix 2× (Thermo Fisher Scientific™, USA), 0.75 µL of Primer Forward (10 µM), 0.75 µL of Primer Reverse (10 µM), and 9 µL of nuclease-free water (Thermo Fisher Scientific™, USA) [78]. Reactions were carried out in a GeneAmp PCR System 9700 thermal cycler (Applied Biosystems, Foster City, CA, USA). Initial denaturation was performed at 94 °C for 5 min; 35 cycles, with an amplification cycle at 94 °C for 30 s, annealing at 52 °C for 30 s, and extension at 72 °C for 30 s; a final elongation at 72 °C for 5 min was added [78].

PCR amplicons were visualized on an UltraPure™ Agarose gel (Thermo Scientific, USA) in 1× UltraPure™ 10× TBE buffer (Thermo Scientific, USA), prestained with UltraPure™ Ethidium Bromide (Invitrogen, Carlsbad, CA, USA), under UV transillumination. Electrophoresis was performed at 100 volts for 62 min.

### 4.5. Genome Characterization

Whole genome sequencing was performed on all *E. coli* isolates with a colistin MIC > 2 μg/mL. It was performed by Illumina 150 paired-end sequencing on a NovaSeq 600 platform (Illumina, Inc., San Diego, CA, USA). Raw reads were quality filtered by Trimmomatic v0.36 [79] and filtered reads were assembled with SPAdes v3.13.0 [80], using default parameters (BioProject PRJNA1274168).

The MLST tool (https://github.com/tseemann/mlst, accessed on 20 December 2024) and SerotypeFinder v.2.0 from the Center for Genomic Epidemiology (CGE) (http://www.genomicepidemiology.org, accessed on 20 December 2024) were used to determine the sequence types and serotypes, respectively, of the *E. coli* genomes [81]. The EzClermont tool (https://nickp60.github.io/EzClermont/, accessed on 3 January 2025) was used to predict phylogroups [82]. All tools were employed using default settings.

Virulence genes were annotated using the CGE VirulenceFinder database with ABRICATE v.1.0.1 tool (https://github.com/tseemann/abricate, accessed on 10 January 2025), while CGE ResFinder v4.6.0 (http://www.genomicepidemiology.org, accessed on 20 August 2024) and the CARD database (https://card.mcmaster.ca/, accessed on 20 August 2024) were used to detect antimicrobial resistance genes, considering 80% coverage and identity cutoffs. Finally, plasmid types were predicted using the Plasmidfinder v2.1 web server [83] (https://cge.food.dtu.dk/services/PlasmidFinder/, accessed on 21 September 2024).

### 4.6. cgMLST Hierarchical Clustering Analysis

Genome Profiler v1.0.1 (https://github.com/jizhang-nz/fast-GeP, accessed on 30 July 2024) was used for cgMLST analysis using *E. coli* str. K-12 substr. MG1655 (NCBI: txid511145) as reference genome [84,85]. Genes were concatenated into a FASTA file using the Ruby script extract_concat_cgMLST_genes.rb (https://github.com/JoseCoboDiaz/concat_cgMLST_genes, accessed on 20 July 2024) for phylogenetic tree construction. This file was used for alignment, and a phylogenetic tree was constructed with MAFFT version 7 [86] using the neighbor-joining method and default parameters for alignment [87], the Jukes-Cantor substitution model and a 1000 bootstrap resampling. The ‘ape’ and the ‘ggtree’ R-packages were used to read the Newick files and plot the phylogenetic tree, respectively. The phylogenetic tree was edited to its final version using InkScape software (https://inkscape.org/).

### 4.7. Statistical Analysis

The data were analyzed using Stata version 14.0 (StataCorp, College Station, TX, USA). Descriptive statistics were used to determine the frequencies of resistant *E. coli* strains for each species (flies, cattle, and pigs). The chi-square test was used to determine the significance of the differences observed between the three species and the multidrug-resistant *E. coli* strains.

## 5. Conclusions

This study observed that the prevalence of multidrug resistance to antimicrobials in *E. coli* strains isolated from cattle feces in a slaughterhouse was low compared to pig and housefly feces.

The genomes of multidrug-resistant and phenotypically colistin-resistant *E. coli* strains isolated from cattle, pigs, and flies displayed chromosomal point mutations linked to colistin resistance. Furthermore, colistin-resistant *E. coli* strains were shown to have plasmid contigs containing a variety of resistance genes.

Our findings show that cattle, pigs, and flies can be reservoirs of colistin-resistant *E. coli* strains, and the house fly is a potential disseminator of antimicrobial-resistant genes from the slaughterhouse to other environments. Furthermore, ongoing surveillance using a One Health approach is urgently needed to monitor the emergence and spread of antimicrobial-resistance genes.

## Figures and Tables

**Table 1:** Antimicrobial resistance prevalence of *Escherichia coli* strains using the Kirby–Bauer method.

Antibiotics	Cattle (%) n = 150	Pig (%) n = 150	Fly (%) n = 150
Lincomycin	100	100	100
Enrofloxacin	11.81	38.62	40.19
Tetracycline	81.89	97.24	90.65
Neomycin	8.66	53.1	49.53
Ampicillin	18.9	89.66	74.77
Amoxicillin	20.47	89.66	72.9
Chloramphenicol	22.05	92.41	69.16
Nalidixic Acid	23.62	70.34	63.55
Sulfatrimethoprim	16.54	77.93	63.55
Colistin	0.79	1.38	7.48
Ciprofloxacin	4.72	31.72	39.25
Nitrofurantoin	0	1.38	3.74
Cephalexin	4.72	6.9	30.84
Gentamicin	6.3	27.59	32.71
Multidrug resistance	19.68	93.73	71.96

**Table 2:** Antimicrobial resistance profile of colistin-resistant *Escherichia coli* strains.

Antibiotics	Cattle	Pig		Fly							
	131V	150B	203B	21M	61M	65M	81M	84M	95M	107M	110M
Lincomycin	R	R	R	R	R	R	R	R	R	R	R
Enrofloxacin	R	R	R	R	S	R	S	S	S	R	S
Tetracycline	R	R	R	R	R	R	R	R	R	R	R
Neomycin	R	R	R	R	R	R	R	R	R	R	R
Ampicillin	S	R	R	R	S	R	R	R	S	R	S
Amoxicillin	S	R	R	S	S	S	S	R	S	R	S
Chloramphenicol	R	R	R	R	R	R	R	R	R	R	R
Nalidixic Acid	R	R	R	R	R	R	R	R	R	R	R
Sulfatrimethoprim	S	R	R	R	S	S	S	R	S	R	S
Ciprofloxacin	R	R	R	R	R	R	R	R	R	R	R
Nitrofurantoin	S	S	S	S	S	S	S	S	S	S	S
Cephalexin	S	R	R	S	S	S	S	S	S	S	S
Gentamicin	R	R	R	R	R	R	R	R	R	R	R

R: resistant; S: susceptible.

**Table 3:** Summary of the characterization of 11 colistin-resistant *E. coli* isolates (sequence, serotype and phylogroup).

Strain	Species	ST	Serotype	Phylogroup	Clonal Complex
107M	fly	-	O13 H29	B1	-
110M	fly	ST 1408	O13 H30	A	-
131V	cattle	ST 937	O43 H2	B1	-
150B	pig	ST 1115	O102 H40	A	-
203B	pig	-	O102 H20	A	-
21M	fly	ST 201	O3 H19	B1	ST469 Cplx
61M	fly	ST 1252	O13 H11	B1	-
65M	fly	ST 6333	O123 H14	B1	-
81M	fly	ST 278	H7	B1	ST278 Cplx
84M	fly	ST 5487	H25	B1	-
95M	fly	ST 351	O18 H7	B1	-

**Table 4:** Virulence genes of 11 colistin-resistant *E. coli* strains isolated from flies, cattle and pigs.

Genes	Isolates										
	Fly								Cattle	Pig	
	107M	110M	21M	61M	65M	81M	84M	95M	131V	150B	203B
*algW*	^+^	^+^	^+^	^+^	^+^	^+^	^+^	^+^	^+^	^+^	^+^
*aslA*	^+^	^+^	^+^	^+^	^+^	^+^	^+^	^+^	^+^	^+^	^+^
*cheA*	^−^	^−^	^+^	^+^	^+^	^+^	^+^	^+^	^+^	^−^	^−^
*cheD*	^+^	^+^	^+^	^+^	^+^	^+^	^+^	^+^	^+^	^+^	^+^
*cheY*	^+^	^+^	^+^	^+^	^+^	^+^	^+^	^+^	^+^	^+^	^+^
*csgB*	^+^	^+^	^+^	^+^	^+^	^+^	^+^	^+^	^+^	^+^	^+^
*csgD*	^+^	^+^	^+^	^+^	^+^	^+^	^+^	^+^	^+^	^+^	^+^
*csgE*	^+^	^+^	^+^	^+^	^+^	^+^	^+^	^+^	^+^	^+^	^+^
*csgF*	^+^	^+^	^+^	^+^	^+^	^+^	^+^	^+^	^+^	^+^	^+^
*csgG*	^+^	^+^	^+^	^+^	^+^	^+^	^+^	^+^	^+^	^+^	^+^
*entA*	^+^	^+^	^+^	^+^	^+^	^+^	^+^	^+^	^+^	^+^	^+^
*entB*	^+^	^+^	^+^	^+^	^+^	^+^	^+^	^+^	^+^	^+^	^+^
*entC*	^+^	^+^	^+^	^+^	^+^	^+^	^+^	^+^	^+^	^+^	^+^
*entD*	^+^	^+^	^+^	^+^	^+^	^+^	^+^	^+^	^+^	^+^	^+^
*entE*	^+^	^+^	^+^	^+^	^+^	^+^	^+^	^+^	^+^	^+^	^+^
*entF*	^+^	^+^	^+^	^+^	^+^	^+^	^+^	^+^	^+^	^+^	^+^
*entS*	^+^	^+^	^+^	^+^	^+^	^+^	^+^	^+^	^+^	^+^	^+^
*espL1*	^+^	^+^	^+^	^+^	^+^	^+^	^+^	^+^	^+^	^+^	^+^
*espL4*	^+^	^+^	^+^	^+^	^+^	^+^	^+^	^+^	^+^	^+^	^+^
*espR1*	^+^	^+^	^+^	^+^	^+^	^+^	^+^	^+^	^+^	^+^	^+^
*espX1*	^+^	^+^	^+^	^+^	^+^	^+^	^+^	^+^	^+^	^+^	^+^
*espX4*	^+^	^+^	^+^	^+^	^+^	^+^	^+^	^+^	^+^	^+^	^+^
*espX5*	^+^	^+^	^+^	^+^	^+^	^+^	^+^	^+^	^+^	^+^	^+^
*espR4*	^+^	^+^	^−^	^−^	^−^	^−^	^−^	^−^	^−^	^−^	^−^
*espX6*	^+^	^−^	^−^	^−^	^−^	^−^	^−^	^−^	^−^	^−^	^−^
*espY2*	^+^	^−^	^−^	^−^	^−^	^−^	^−^	^−^	^−^	^−^	^−^
*espY4*	^+^	^−^	^−^	^−^	^−^	^−^	^−^	^−^	^−^	^−^	^−^
*fdeC*	^+^	^+^	^+^	^+^	^+^	^+^	^+^	^+^	^+^	^+^	^+^
*fepA*	^+^	^+^	^+^	^+^	^+^	^+^	^+^	^+^	^+^	^+^	^+^
*fepB*	^+^	^+^	^+^	^+^	^+^	^+^	^+^	^+^	^+^	^+^	^+^
*fepC*	^+^	^+^	^+^	^+^	^+^	^+^	^+^	^+^	^+^	^+^	^+^
*fepD*	^+^	^+^	^+^	^+^	^+^	^+^	^+^	^+^	^+^	^+^	^+^
*fepG*	^+^	^+^	^+^	^+^	^+^	^+^	^+^	^+^	^+^	^+^	^+^
*f17d-A*	^−^	^−^	^−^	^−^	^−^	^−^	^−^	^−^	^+^	^−^	^−^
*f17d-C*	^−^	^−^	^−^	^−^	^−^	^−^	^−^	^−^	^+^	^−^	^−^
*f17d-D*	^−^	^−^	^−^	^−^	^−^	^−^	^−^	^−^	^+^	^−^	^−^
*f17d-G*	^−^	^−^	^−^	^−^	^−^	^−^	^−^	^−^	^+^	^−^	^−^
*fes*	^+^	^+^	^+^	^+^	^+^	^+^	^+^	^+^	^+^	^+^	^+^
*fimA*	^+^	^+^	^+^	^+^	^+^	^+^	^+^	^+^	^+^	^+^	^+^
*fimB*	^+^	^+^	^+^	^+^	^+^	^+^	^+^	^+^	^+^	^+^	^+^
*fimC*	^+^	^+^	^+^	^+^	^+^	^+^	^+^	^+^	^+^	^+^	^+^
*fimD*	^+^	^+^	^+^	^+^	^+^	^+^	^+^	^+^	^+^	^+^	^+^
*fimE*	^+^	^+^	^+^	^+^	^+^	^+^	^+^	^+^	^+^	^+^	^+^
*fimF*	^+^	^+^	^+^	^+^	^+^	^+^	^+^	^+^	^+^	^+^	^+^
*fimG*	^+^	^+^	^+^	^+^	^+^	^+^	^+^	^+^	^+^	^+^	^+^
*fimH*	^+^	^+^	^+^	^+^	^+^	^+^	^+^	^+^	^+^	^+^	^+^
*fimI*	^+^	^+^	^+^	^+^	^+^	^+^	^+^	^+^	^+^	^+^	^+^
*flgD*	^+^	^−^	^+^	^−^	^+^	^+^	^−^	^+^	^+^	^−^	^−^
*flgF*	^+^	^−^	^−^	^−^	^−^	^−^	^−^	^−^	^−^	^−^	^−^
*flgE*	^+^	^+^	^+^	^+^	^+^	^+^	^+^	^+^	^+^	^+^	^+^
*flgG*	^+^	^+^	^+^	^+^	^+^	^+^	^+^	^+^	^+^	^+^	^+^
*flgH*	^+^	^+^	^+^	^+^	^+^	^+^	^+^	^+^	^+^	^+^	^+^
*flgM*	^+^	^+^	^+^	^+^	^+^	^+^	^+^	^+^	^+^	^+^	^+^
*flgJ*	^+^	^+^	^−^	^+^	^−^	^−^	^+^	^−^	^−^	^+^	^+^
*flhA*	^+^	^+^	^+^	^+^	^+^	^+^	^+^	^+^	^+^	^+^	^+^
*fliG*	^+^	^+^	^+^	^+^	^+^	^+^	^+^	^+^	^+^	^+^	^+^
*fliI*	^+^	^+^	^+^	^+^	^+^	^+^	^+^	^+^	^+^	^+^	^+^
*fliM*	^+^	^+^	^+^	^+^	^+^	^+^	^+^	^+^	^+^	^+^	^+^
*fliN*	^+^	^+^	^+^	^+^	^+^	^+^	^+^	^+^	^+^	^+^	^+^
*fliP*	^+^	^+^	^+^	^+^	^+^	^+^	^+^	^+^	^+^	^+^	^+^
*fliC*	^+^	^−^	^−^	^+^	^−^	^−^	^+^	^−^	^+^	^+^	^+^
*gmd*	^+^	^−^	^−^	^−^	^−^	^−^	^−^	^−^	^−^	^−^	^−^
*gspC*	^−^	^−^	^+^	^+^	^+^	^+^	^+^	^−^	^+^	^+^	^+^
*gspD*	^−^	^−^	^+^	^+^	^+^	^+^	^+^	^−^	^+^	^+^	^+^
*gspE*	^−^	^−^	^+^	^+^	^+^	^+^	^+^	^−^	^+^	^+^	^+^
*gspF*	^−^	^−^	^+^	^+^	^+^	^+^	^+^	^−^	^+^	^+^	^+^
*gspG*	^+^	^+^	^+^	^+^	^+^	^+^	^+^	^+^	^+^	^+^	^+^
*gspH*	^−^	^−^	^+^	^+^	^+^	^+^	^+^	^+^	^+^	^+^	^+^
*gspI*	^−^	^−^	^+^	^+^	^+^	^+^	^+^	^+^	^+^	^+^	^+^
*gspJ*	^+^	^+^	^+^	^+^	^+^	^+^	^+^	^+^	^+^	^+^	^+^
*gspK*	^+^	^+^	^+^	^+^	^+^	^+^	^+^	^+^	^+^	^+^	^+^
*gspL*	^+^	^+^	^+^	^+^	^+^	^+^	^+^	^+^	^+^	^+^	^+^
*gspM*	^+^	^+^	^+^	^+^	^+^	^+^	^+^	^+^	^+^	^+^	^+^
*gtrA*	^+^	^+^	^−^	^+^	^−^	^−^	^−^	^−^	^−^	^−^	^−^
*gtrB*	^+^	^+^	^−^	^+^	^−^	^−^	^−^	^−^	^−^	^−^	^−^
*gtrII*	^+^	^+^	^−^	^−^	^−^	^−^	^−^	^−^	^−^	^−^	^−^
*katB*	^+^	^+^	^−^	^+^	^+^	^+^	^+^	^+^	^+^	^+^	^+^
*ompA*	^+^	^+^	^+^	^+^	^+^	^+^	^+^	^+^	^+^	^+^	^+^
*sfaF*	^+^	^−^	^−^	^−^	^−^	^−^	^−^	^−^	^−^	^−^	^−^
*pefC*	^−^	^−^	^+^	^−^	^−^	^−^	^−^	^−^	^−^	^−^	^−^
*pefD*	^−^	^−^	^+^	^−^	^−^	^−^	^−^	^−^	^−^	^−^	^−^
*iroB*	^−^	^−^	^−^	^−^	^−^	^−^	^+^	^−^	^−^	^−^	^−^
*iroC*	^−^	^−^	^−^	^−^	^−^	^−^	^+^	^−^	^−^	^−^	^−^
*iroD*	^−^	^−^	^−^	^−^	^−^	^−^	^+^	^−^	^−^	^−^	^−^
*iroE*	^−^	^−^	^−^	^−^	^−^	^−^	^+^	^−^	^−^	^−^	^−^
*iroN*	^−^	^−^	^−^	^−^	^−^	^−^	^+^	^−^	^−^	^−^	^−^
*yagV/ecpE*	^+^	^+^	^+^	^+^	^+^	^+^	^+^	^+^	^+^	^+^	^+^
*yagW/ecpD*	^+^	^+^	^+^	^+^	^+^	^+^	^+^	^+^	^+^	^+^	^+^
*yagX/ecpC*	^+^	^+^	^+^	^+^	^+^	^+^	^+^	^+^	^+^	^+^	^+^
*yagY/ecpB*	^+^	^+^	^+^	^+^	^+^	^+^	^+^	^+^	^+^	^+^	^+^
*yagZ/ecpA*	^+^	^+^	^+^	^+^	^+^	^+^	^+^	^+^	^+^	^+^	^+^
*ykgK/ecpR*	^+^	^+^	^+^	^+^	^+^	^+^	^+^	^+^	^+^	^+^	^+^
*irp1*	^−^	^−^	^−^	^+^	^−^	^−^	^−^	^−^	^−^	^−^	^−^
*irp2*	^−^	^−^	^−^	^+^	^−^	^−^	^−^	^−^	^−^	^−^	^−^
*fyuA*	^−^	^−^	^−^	^+^	^−^	^−^	^−^	^−^	^−^	^−^	^−^

(+): presence; (−): absence.

## Data Availability

The data presented in this study are available upon request from the corresponding author.
